# Resistance Training with Blood Flow Restriction and Hypertensive Subjects

**DOI:** 10.1515/hukin-2015-0028

**Published:** 2015-07-10

**Authors:** Iván Chulvi-Medrano

**Affiliations:** 1 Sport Sciences, CSCS, NSCA-CPT*; Benestar Wellness Center Research.

***Dear Editor-in-Chief,***

A recent article published in the Journal of Human Kinetics (Aráujo et al., 2014) described a post-exercise hypotensive effect in hypertensive subjects after resistance exercise with blood flow restriction. The aim of this letter is to endorse the importance of further research on post-exercise hypotensive effect as a result of resistance training with blood flow restriction, especially in hypertensive subjects.

Resistance exercise with blood flow restriction (REBFR) is considered alternative training for enhancing strength and muscle size (hypertrophy) to high intensity conventional resistance training (Pope et al., 2013). It is often recommended to include REBFR in clinical population, including hypertension (HTA), as this type of training carries a lower risk of injury. Additionally, it has been reported that REBFR is safe (Nakajima et al., 2006; Loenneke et al., 2011). For instance, Nakajima et al. (2006) examined the incidence of adverse events of this new methodology using a survey on 12.642 subjects that had received resistance exercise with blood flow restriction. In this report only 2 cases of increasing blood pressure were detected. The author’s conclusion stated that resistance exercise with blood flow restriction was safe including clinical population such as hypertension (Nakajima et al., 2006). The Journal of Human Kinetics has recently published an article in which 14 type-1 hypertensive women were enrolled in knee extension exercise, 3 sets of 15 repetitions. One group used blood flow restriction (80% of arterial occlusion pressure) with a 30% RM intensity and the other group trained without occlusion using 80% 1RM. Researchers monitored blood pressure after 60 minutes and found that the occlusive group experienced a significant post-exercise hypotensive effect (PEHE). These data are very promising because they show that a PEHE after REBFR in HTA, an effect that has already been noticed previously in normotensive subjects (Benito and Chulvi, 2013), exists. We found that the intensity was a meaningful variable, taking into account that 70% 1RM decreased the blood pressure more than 30% (Benito and Chulvi, 2013). In hypertensive subjects, high intensity resistance training is contraindicated, but muscular strength is an important component of physical fitness. According to this, applying blood flow restriction in resistance training may be an excellent choice. On the other hand, little evidence has been accumulated supporting the hypothesis of a PEHE after REBFR. It is important to highlight that research in a PEHE after REBFR is not consistent in normotensive subjects. For instance, Rossow et al. (2011) did not find a PEHE after REBFR performing 20% 1RM for lower extremity.

Therefore, further investigation is required to assess the physiological effects on hypertensive subjects comparing: a) resistance training with different levels of blood flow restriction; b) different intensities in resistance training; c) REBFR versus traditional resistance training; d) REBFR versus traditional aerobic training in hypertensive subjects.
